# Enzyme-treated soybean meal in diets for weanling pigs: review

**DOI:** 10.5713/ab.250731

**Published:** 2026-07-27

**Authors:** Leidy J. Torres-Mendoza, Hans H. Stein

**Affiliations:** 1 Department of Animal Sciences, University of Illinois, Urbana, IL, USA

**Keywords:** Anti-nutritional Factor, Digestibility, Enzyme-treated Soybean Meal, Soybean Meal, Weanling Pigs

## Abstract

The objective of the present contribution is to provide a summary of the nutritional value of enzyme-treated soybean meal (ESBM) and review effects of ESBM on growth performance of weanling pigs. Guidelines for inclusion rates of ESBM in diets for weanling pigs will also be provided based on the published literature. The literature review was conducted in May 2024 by searching Google Scholar and PubMed for experiments in which ESBM was included in diets for weanling pigs, using Boolean queries implemented with Biopython/Entrez. A total of 24 unique papers were identified. These papers were manually reviewed to verify their relevance to the specific topics of composition, digestibility, and health/performance outcomes. Enzyme-treated soybean meal contains 48.3% to 57.7% crude protein, is rich in amino acids, and has elevated small-peptide fractions. Anti-nutritional factors such as raffinose and stachyose were reduced by 50% and 90%, respectively, compared with conventional soybean meal (SBM), and trypsin inhibitors are reduced to 1.7 units per mg. Enzyme-treated SBM also contains more fiber, but less monosaccharides and oligosaccharides, than SBM. Enzyme-treated SBM was associated with fewer gastrointestinal disturbances, favorable changes in intestinal microbiota, and improved intestinal barrier indices. In most experiments, pigs fed ESBM had improved growth performance and less diarrhea compared with pigs fed diets containing conventional SBM. Most experiments that examined the nutritional value of ESBM concluded that ESBM supports improved nutrient utilization, growth performance, and health in weanling pigs and on average, inclusion rates of 4% to 10% is recommended. In conclusion, Enzyme-treated SBM has greater concentration of protein and contains fewer anti-nutritional factors than conventional SBM, which results in greater intestinal health and improved growth performance of weanling pigs if the inclusion rate of ESBM is 4% to 10% in diets for newly weaned pigs.

## INTRODUCTION

Soybeans is one of the major agricultural crops in the world, and global annual production is close to 400 million metric tons [1]. The three major producers of soybeans in the world are Brazil, the United States, and Argentina, which produce 39%, 29%, and 13%, respectively, of global production [1]. In 2023, soybean production in the United States was around 115 million metric tons, and Illinois was the state with the most hectares planted with soybeans [1,2]. Most soybeans are crushed to provide soybean oil and soybean meal (SBM) [3]. Soybean oil is used in food applications, biodiesel production, or production of other industrial products, but most SBM is used as a source of amino acids (AA) in diets for pigs, poultry, dairy, and other animals.

Soybean meal produced in the United States often has a greater concentration of AA compared with SBM from China or Argentina [4,5], but regardless of origin, SBM contains all the indispensable AA needed by pigs and poultry. Specifically, the concentration of lysine and tryptophan is greater in SBM than in most other plant proteins. Conventional dehulled and toasted SBM is produced by removing the fat from dehulled soybeans using a solvent. After this procedure, a toasting step is needed to reduce trypsin inhibitors and lectins [6–8] and to remove residual solvent [9]. The resulting SBM is an excellent source of AA for growing-finishing pigs and reproducing sows, but because of antigens and oligosaccharides [6,10], SBM cannot be used as the only source of AA in diets for weanling pigs [11]. Therefore, diets for weanling pigs have traditionally contained animal proteins (i.e., fish meal, milk protein, blood protein) in addition to limited quantities of conventional SBM. However, due to increased demand for animal proteins from other industries, the supply of animal proteins for pig feed has been reduced over the last few decades.

If conventional SBM is inoculated with yeast and enzymes, both antigens and oligosaccharides can be eliminated [9]. The resulting product is called enzyme-treated soybean meal (ESBM) and is produced by treating dehulled, solvent-extracted SBM for several hours with yeast and a proprietary blend of enzymes [12]. Enzymes in the mixture include pectinase, alpha-galactosidase, cellulase, and sucrase [13], and the degree to which antigens and oligosaccharides are inactivated during the process is influenced by the enzyme mixture, fermentation conditions, time, temperature, and pH. Due to the reduced concentrations of oligosaccharides and antigens, ESBM may be used in diets for weanling pigs [9].

The nutritional composition of ESBM including the concentration of anti-nutritional factors (ANF) has been reported [6,12–14]. The impact of adding ESBM to diets for weanling pigs has also been determined [11,14–17] and effects of ESBM on intestinal health of weanling pigs have been investigated [14,18–20]. However, no comprehensive reviews covering all aspects of ESBM fed to weanling pigs are available and the optimal inclusion rate of ESBM in diets for weanling pigs has not been reported. Likewise, there are no published feeding guidelines for ESBM based on published data.

The objective of the present contribution is, therefore, to provide a summary of the nutritional value of ESBM, and review effects of ESBM on growth performance and immune status of weanling pigs. Guidelines for inclusion rates of ESBM in diets for weanling pigs will also be provided based on the published literature. However, it is outside the scope of this review to discuss management practices, housing conditions, and health status of pigs, and how these factors may influence growth performance and immune responses independently of ESBM inclusion.

## MATERIALS AND METHODS

The literature review was conducted in May 2024 by searching PubMed and Google Scholar. In Google Scholar databases, the search was conducted using “ESBM and weanling pigs” and 189 publications were initially identified, but after examination, only 23 publications were selected. In PubMed (MEDLINE), queries targeted ESBM in weanling pigs and were designed to retrieve studies on composition, digestibility, and health/performance outcomes [21].

To ensure reproducibility of the literature search, a custom Python script was developed using Biopython v1.81 [22] and executed in Python 3.11.4 within a Jupyter Notebook. The script used the Entrez module to query PubMed with a Boolean search combining ESBM-related terms, weanling pig identifiers, and keywords related to composition, digestibility, and performance. The query returned a list of PubMed IDs, from which metadata were retrieved and exported to Excel using the pandas library. This automated workflow enabled standardized, transparent search documentation, thereby addressing the need for reproducible data mining, consistent with the bioinformatics tools cited [22]. The code assembled: (i) an ESBM term set (e.g., “enzyme-treated soybean meal”, “enzymolytic soybean meal”, “enzymatically treated soybean meal”, “enzyme-modified soybean meal”, ESBM, “HP300”, “HP310”, plus spacing variants), (ii) a porcine/weanling term set (e.g., “weanling pig*”, “nursery pig*”, piglet*, porcine, swine, *Sus scrofa*), and (iii) three thematic term sets (composition, digestibility, health/performance) that included common synonyms (e.g., “nutrient/chemical composition”, “ileal/total tract/amino acid digestibility”, “gut health”, “intestinal morphology”, “immune/oxidative/diarrhea/growth performance”). This final code was entered into PubMed as the search query: ((“enzyme-treated soybean meal”[TIAB] OR “enzymolytic soybean meal”[TIAB] OR “enzymatically treated soybean meal”[TIAB] OR “enzyme-modified soybean meal”[TIAB] OR ESBM[TIAB] OR “HP300”[TIAB] OR “HP310”[TIAB]) AND (“weanling pig*”[TIAB] OR “nursery pig*”[TIAB] OR piglet*[TIAB] OR porcine[TIAB] OR swine[TIAB] OR “Sus scrofa”[MeSH Terms]) AND (composition[TIAB] OR “nutrient composition”[TIAB] OR “chemical composition”[TIAB] OR digestibility[TIAB] OR “amino acid digestibility”[TIAB] OR “ileal digestibility”[TIAB] OR “gut health”[TIAB] OR “intestinal morphology”[TIAB] OR “immune response”[TIAB] OR diarrhea[TIAB] OR “growth performance”[TIAB])).

The selection of papers for the review followed a systematic process. Initially, papers were identified by their titles to determine relevance to the topic of ESBM in weanling pigs. Each final query required at least one ESBM term, one porcine/weanling term, and one theme term, with appropriate field tags used for PubMed. The code then searched each individual query on PubMed MEDLINE and returned an output file (i.e., a Microsoft Excel workbook) containing 22 results.

Two of these papers were removed based on their titles; thus, a total of 43 publications were initially selected from the two databases. However, because many publications appeared in more than one database, only 24 unique publications were retained. These papers were manually reviewed by reading to verify their relevance to the specific topics of composition, digestibility, and health/performance outcomes. A table was created to document the key findings from each selected paper, including study outcomes, methodologies, and results. The information was then organized for clarity and consistency, with simple statistical analyses conducted to summarize and highlight patterns across the selected studies. The baseline characteristics of the publications that met the final criteria are summarized in Table 1 and Figure 1.

### Statistical analysis

Basic descriptive statistics were calculated when multiple values were available for each parameter. The average was used to represent the overall trend, while the standard deviation (SD) and coefficient of variation (CV) were included to reflect the variability in the data. This approach allowed for a summary of the literature and helped highlight both patterns and discrepancies among reported results.

## RESULTS AND DISCUSSION

### Composition of enzyme-treated soybean meal

Enzyme treatment of SBM results in an increase in the concentration of crude protein (CP), AA, ether extract (EE), ash, neutral detergent fiber (NDF), crude fiber, and minerals compared with conventional SBM [10,12]. The reason for this increase is that fermentation of oligosaccharides and sucrose during the production of ESBM eliminates these components, which concentrates the other nutrients in the final product (Tables 2, 3; Figure 2).

Enzyme-treated soybean meal also has slightly greater concentration of dry matter (DM) than conventional SBM because of the drying step that is needed after inoculation of the SBM with enzymes. However, the CV of 2.0% indicates that the moisture content is relatively stable among samples [23–27]. The concentration of acid-hydrolyzed ether extract (AEE) in ESBM is only 1.7% on average and as in the case for other defatted soybean products, ESBM has low concentration of AEE [10].

The concentration of CP in ESBM is 54.2% on average with a range from 48.3% to 57.7%. The high protein concentration is one of the main reasons ESBM is a valuable ingredient in animal diets. The variation in concentration of CP may be due to differences in CP among batches of SBM used for ESBM production [6,11,12]. The concentration of AA in ESBM reflects the profile in SBM and generally is high in indispensable AA, including lysine and tryptophan [23,28–31]. The CV for the concentration of the indispensable AA ranges from 19.1% to 31.8%, which indicates some differences among samples, which may be due to differences in the SBM used in the production of ESBM. However, the CV for most AA is greater in ESBM than in conventional SBM, which indicates that variation among sources of ESBM is partly a result of variability in processing conditions [32–34]. The lower CV observed for dispensable AA than indispensable AA in ESBM is likely a result of the potential for heat damage of some indispensable AA including arginine, lysine and methionine. These AA are more susceptible to heat damage than most of the dispensable AA. The high CV for tryptophan may be a result of inconsistent hydrolysis during the alkaline digestion, which can result in differences in analyzed values. Likewise, sulfur-containing AA (i.e., methionine and cysteine), require performic acid oxidation before acid hydrolysis, which may also introduce variability, and therefore, contribute to the greater CV observed for indispensable AA compared with dispensable AA.

Concentrations of calcium and phosphorus in ESBM are 0.32% and 0.72%, respectively. However, other minerals including chlorine, potassium, magnesium, sodium, sulfur, chromium, copper, iron, iodine, manganese, selenium, and zinc are not reported [34–38]. Although the calcium and phosphorus concentrations of ESBM are similar to those of SBM [10], no information is available on whether the concentrations of other minerals are also similar. Additional research is needed to characterize the mineral composition of ESBM.

Enzyme-treated SBM has a low concentration of glucose, maltose, fructose, sucrose, and starch compared with conventional SBM, which is likely because these compounds are fermented during the production of ESBM. The average concentration of crude fiber is 3.84%, but the CV of 25.19% indicates that fiber can vary among batches of ESBM, which may be due to differences among sources in raw material.

Neutral detergent fiber represents the hemicellulose, cellulose, and lignin in the fiber and ESBM contains 11.92% NDF, and the CV is almost 20% indicating that there is quite a bit of variability among sources of ESBM in NDF. Acid detergent fiber (ADF) includes cellulose and lignin, and ADF in ESBM is 5.74%. Compared with conventional SBM, NDF is generally greater in ESBM [10,12,34], indicating that fiber is not degraded or fermented during the enzymatic processing used to produce ESBM. This increase in NDF may result from the accumulation of hemicellulose or the formation of fiber-like polysaccharide complexes, potentially from Maillard reaction products or microbial residues, especially in fermented products. In contrast, reported changes in ADF are inconsistent among experiments with some values being greater for ESBM [10,12,34], whereas others have reported decreased ADF in ESBM [11,16]. However, it is not clear if the same batch of SBM was always analyzed before and after enzymatic treatment, making it difficult to attribute changes in ADF directly to the processing. The greater consistency of NDF increases compared with ADF supports the idea that most of the fiber-related changes occur in the hemicellulose fraction, which is included in NDF, but not in ADF.

The total dietary fiber (TDF) combines both insoluble dietary fiber (IDF) and soluble dietary fiber (SDF). Insoluble dietary fiber in ESBM is 18.97%, whereas SDF is 2.06%. The TDF in ESBM is 21.02% on average, which is greater than in conventional SBM, further indicating that fiber is not lost during fermentation and enzymatic treatment. The CV of TDF and IDF in ESBM is less than 20%, but because of the low concentration of SDF, the CV for SDF is much greater than the CV for TDF and IDF. The observation that TDF was greater than NDF may be attributed to the SDF that is included in TDF, but not in NDF. However, IDF was also greater than NDF, which indicates that the NDF analysis may not capture all the insoluble fiber in ESBM.

Soybean meal contains oligosaccharides [16], trypsin inhibitors, lectins, urease, and allergenic proteins [8], which may reduce AA digestibility [16] and cause gastrointestinal disturbances, which may negatively affect growth performance of young pigs [8,29]. However, in ESBM, the concentration of sucrose and stachyose is reduced by approximately 94% and raffinose by around 50% compared with conventional SBM [6,11,12,16]. Because oligosaccharides have a negative effect on growth performance of weanling pigs and can cause increased diarrhea [39], the reduction in oligosaccharides in ESBM may positively impact growth of pigs.

On average, the concentration of trypsin inhibitors and lectins in ESBM is 1.7 TIU/mg and 29 mg/kg, respectively, which is less than in conventional SBM [7,27]. This reduction may be due to the fermentation and the drying after the fermentation. The reduction in trypsin inhibitors is also evidenced by a reduction in urease [8]. Due to the use of enzymes in the production of ESBM, the concentration of glycinin and β-conglycinin is reduced compared with conventional SBM [6,14,18]. The low concentration of trypsin inhibitors, glycinin, β-conglycinin, and oligosaccharides confirms that fermentation and enzyme treatment is an effective method for reducing these ANF.

Small or short peptides are defined as peptides with at least two and not more than 45 AA [6] and ESBM has a greater concentration of small peptides compared with conventional SBM (Table 4) because of the enzymatic reaction during production [8,16,18,26]. Small peptides are believed to be more easily absorbed from the small intestine and they may also improve the beneficial gut microbiota community [29,31,40]. Increasing the concentration of small peptides may increase digestibility of AA and may also increase the speed of absorption of AA [30,35,40,41]. As a consequence, pigs fed a diet containing ESBM had a greater plasma appearance of AA than pigs fed conventional SBM during the first two hours after feeding, but three hours after feeding, no differences between the two sources of SBM were observed [30]. These observations indicate that the smaller peptide sizes in ESBM compared with conventional SBM result in a faster uptake of AA, which may enhance protein synthesis, improve nutrient utilization, support gut health and immunity, and contribute to better growth performance and feed efficiency in weaned pigs [30].

### Digestibility of amino acids in enzyme-treated soybean meal

Apparent ileal digestibility (AID) of AA refers to the proportion of dietary AA absorbed at the end of the small intestine, calculated by subtracting the amount of each AA recovered in the ileal digesta from the amount ingested. This measure does not account for endogenous AA losses and may, therefore, underestimate true digestibility. Nevertheless, AID is widely used to determine bioavailability of AA in feed ingredients. In ESBM, the average AID of CP is 77.30%. The CV of AID of AA and CP in ESBM was less than 12.00%, except for proline (Table 5).

The average standardized ileal digestibility (SID) of CP is 87.60% (Table 6). For indispensable AA, the averages indicate that arginine and methionine have SID of 94.40% and 91.60%, respectively. The SID of isoleucine, leucine, and tryptophan is between 89.60 and 90.70%, whereas the SID of lysine and threonine is 86.10% and 85.40%, respectively. Some sources of ESBM have reduced SID of lysine compared with SID of lysine in SBM (89.00%) [10], which may be due to Maillard reaction initiated during drying of ESBM [11,34]. The Maillard reaction causes lysine to bind to reducing sugars, which makes it unavailable for use in protein synthesis by animals. The Maillard reaction reduces both the concentration of analyzed lysine and the SID of lysine [6].

The SID of proline in ESBM is 94.80% on average, indicating a high digestibility compared with all other AA. The SID of tyrosine and glutamic acid is 91.20% and 89.40%, respectively, whereas the SID of glycine and cysteine is 83.50% and 80.00% respectively. The CV for the SID of AA and CP in ESBM is less than 9.00%, except for proline. The low CV for the SID of CP and most AA in ESBM indicates moderate variability of the digestibility among experiments.

### Total tract digestibility of energy and nutrients in enzyme-treated soybean meal

The apparent total-tract digestibility (ATTD) of DM is 87.30%, and the ATTD of CP is approximately 80.00%, whereas, the ATTD of gross energy on average is 84.60% (Table 7) [10,11,15,16,18,25]. It appears that enzyme treatment of SBM may increase gross energy in the ingredient, but ATTD of gross energy is not different in ESBM compared with SBM [12].

The ATTD of calcium in ESBM ranges between 53.60% and 63.80%, averaging 58.70%, and ATTD of phosphorus averages 61.80% with a CV of 3.20%. The greater digestibility of phosphorus in ESBM than in conventional SBM (ATTD: 39.00%) [10] is a consequence of the fermentation process that results in the release of phosphorus from the phytate molecule, which makes more phosphorus digestible to pigs. This may result in reduced phosphorus excretion from pigs, and less phosphorus from feed phosphates will be needed in diets containing ESBM, which will contribute to a reduction in diet cost [42,43].

### Effect of enzyme-treated soybean meal on intestinal health

The reduced ANF in ESBM compared with conventional SBM reduces gastrointestinal diseases in pigs with a subsequent increase in the health status of weanling pigs [8]. The increased concentration of small peptides in ESBM may also improve intestinal microbiota and promote the abundance of beneficial bacteria in nursery pigs [29]. Indeed, feeding diets containing ESBM improved intestinal morphology, including increased villus height and villus:crypt ratio, compared with conventional SBM, and other soybean products which may contribute to enhanced AA absorption [16]. Replacing 50% of full-fat soybeans in the diet with ESBM improved intestinal health and reduced diarrhea in pigs because it reduced protein fermentation in the hindgut [19,29].

Feeding pigs with high levels of protein can cause an inflammatory response, which may damage the intestine, reduce the digestibility of nutrients, and increase diarrhea [18,19,29]. Using ESBM in diets decreased the levels of pro-inflammatory cytokines tumor necrosis factor (TNF-α), interferon-γ (IFN-γ), interleukin-1β (IL-1β), and IL-6, which is an indication that inflammation was reduced [18,29]. Additionally, pigs fed a diet containing ESBM had a greater concentration of IgG and IL-10 in serum than pigs fed a diet with conventional SBM, which may be a result of improved immunity of the animal [29]. Despite the high concentration of CP in ESBM, inclusion of ESBM in diets for weanling pigs has consistently been demonstrated to reduce inflammatory responses in weanling pigs. This is likely a consequence of the enzymatic processing of ESBM that reduces concentrations of glycinin, β-conglycinin, and trypsin inhibitors, which likely reduces gut irritation [6]. Additionally, enzymatic hydrolysis hydrolyzes large soy proteins into smaller peptides and free AA that are more easily absorbed in the small intestine, thereby reducing the amount of undigested protein reaching the hindgut, which limits protein fermentation and toxic metabolite formation [29].

Intestinal health may be influenced by the intestinal microbiota, which is highly dependent on the diet [15,20]. Using *Lactobacillus* in diets for pigs may improve growth performance, carcass quality, and intestinal health [44], whereas *Streptococcus* is associated with septicemia and other infections [20]. Pigs fed diets containing ESBM had increased *g_Blautia* (*f_Lachnospiraceae*) and *g_Lactobacillus* compared with pigs fed extruded full-fat soybeans, and the concentration of *Bifidobacterium*, which is beneficial to intestinal health, was greater in the cecum of pigs fed a diet containing ESBM than in pigs fed a diet containing extruded full-fat soybeans [15]. These changes in intestinal microbiota may be the reason pigs fed ESBM had improved intestinal health.

The intestinal barrier is an important protective barrier in the intestines, and an intact barrier reduces the negative impact of pathogens, toxins, and antigens on the mucosa. Pigs fed a diet with ESBM had reduced concentrations of serum diamine oxidase, which indicates that these pigs had an improved gut barrier compared with pigs fed conventional SBM [19,29]. The reduced concentration of the main antigens in ESBM compared with conventional SBM also contributed to reduced diarrhea, improved immunity, improved intestinal barrier function, increased villus height, and increased nutrient digestibility [18,29]. The concentration of the volatile fatty acids acetate, propionate, and butyrate, and total volatile fatty acids in ileal contents increased as ESBM inclusion increased, which may be a consequence of reduced digesta transit rate in the small intestine, resulting in increased fermentation of fiber in the ileum [19].

### Effect of enzyme-treated soybean meal on growth performance of pigs

Pigs fed diets with high levels of conventional SBM have low digestibility and absorption of nutrients, low growth rate, and increased incidence of diarrhea, which is believed to be due to the ANF in SBM [16,17,29]. However, because of the reduced ANF in ESBM, growth rate of pigs may be increased if ESBM is used instead of conventional SBM [6,11,12,29]. Pigs fed diets containing ESBM also have reduced diarrhea compared with pigs fed diets with extruded full-fat SBM, conventional SBM, soy protein concentrate, fermented SBM, or fish meal [8,29,31,36,37].

Enzyme-treated SBM is less expensive than milk proteins and blood proteins, which are sometimes used as AA-containing ingredients in diets for weanling pigs. Pigs fed diets containing ESBM instead of SBM had reduced average daily feed intake, but average daily gain was not affected, resulting in improved gain-to-feed ratio in pigs fed ESBM diets [14,40]. These improvements in feed efficiency were likely due to enhanced nutrient digestibility and reduced ANF in ESBM compared with SBM [12,16,26]. Ruckman et al. [19] included 7%, 14%, or 21% ESBM in diets for weanling pigs, and pigs fed the diets with 14% or 21% ESBM had reduced average daily gain and final body weight compared with pigs fed the diet with 7% ESBM, but the gain-to-feed ratio was not affected. It is, however, possible that the source of ESBM used in the experiment may have been heat-damaged, but because no measures for heat damage were reported, this hypothesis cannot be verified. Regardless, as is the case for any other feed ingredient, it is important that ESBM is not heat-damaged during production or drying because that reduces growth performance [11].

Substituting SBM for 6% to 8% ESBM improved growth performance in weanling pigs by enhancing nutrient digestibility, boosting antioxidant capacity, and strengthening intestinal barrier function [26]. Similarly, dietary inclusion of 8% ESBM reduced the diarrhea index and improved nutrient utilization, contributing to better growth outcomes [7].

Enzyme-treated SBM, when included at 10% to 15% of the diet, along with soy protein concentrate and fermented SBM, enabled a reduction in inclusion of animal protein in diets for pigs without compromising growth or intestinal health. In comparison to fish meal, including 7% to 14% of ESBM, provided equal or improved growth performance and reduced the incidence of diarrhea, and also resulting in better antioxidant capacity and intestinal integrity [27]. Increased average daily gain, improved immune responses, and enhanced antioxidant status in pigs fed diets containing 9% to 12% ESBM have also been reported, which may have been associated with better nutrient absorption and reduced protein fermentation in the hindgut [14,31].

Improved growth performance due to enhanced immune response and improved intestinal morphology was observed when 10% ESBM was included in diets for weanling pigs [29]. Specifically, pigs fed the ESBM diet had an average daily gain of 445 g, whereas pigs fed a diet containing full-fat soybeans gained only 366 g per day [29]. In low-protein diets, inclusion of up to 15% ESBM maintained growth performance and reduced nitrogen excretion compared with pigs fed diets based on fish meal, indicating both nutritional and environmental advantages [37]. Additionally, partial replacement of SBM and soy protein isolate with ESBM improved feed intake, weight gain, and immune function in weanling piglets [8].

### Recommended inclusion rates and feeding guidelines

The recommended inclusion rate for ESBM in diets for weanling pigs is 4% to 10% during the early nursery phase (7 kg to 11 kg body weight). Inclusion of 4% ESBM in the diets instead of SBM resulted in improved average daily gain, villus height, superoxide dismutase, and total antioxidant capacity, and reduced diarrhea [26]. Similarly, increasing ESBM from 5% to 15% increased average daily gain from 279 to 349 g [8]. Pigs fed a diet containing 4%, 7%, or 14% ESBM had greater gain-to-feed ratio compared with pigs fed a control diet without ESBM [14,29,37]. Combined, these results indicate that inclusion of ESBM at 4% to 10% in diets for weanling pigs will improve growth, feed efficiency, and gut health during the early post-weaning period. As pigs transition to a phase 2 diet (11 kg to 25 kg body weight), inclusion rates of 10% to 15% are appropriate, as ESBM can effectively replace animal protein supplements without compromising intestinal health or average daily gain [27,40]. In low-protein, AA-supplemented diets, ESBM may be included by up to 15% without negatively impacting growth performance.

## CONCLUSION

Enzyme-treated SBM has a greater concentration of nutrients and contains fewer ANF than conventional SBM, which results in improved intestinal health and growth performance of weanling pigs. Therefore, ESBM can replace animal proteins in diets for young pigs. It is, however, important to make sure that ESBM is not heat-damaged during production because that will reduce the digestibility of other nutrients, including lysine. Inclusion of 6% to 10% ESBM in starter diets and 10% to 15% in subsequent diets fed during the post-weaning period appears to result in the best growth performance. In low-protein diets, it is possible that slightly greater inclusion rates may be used.

## Figures and Tables

**Figure 1:**
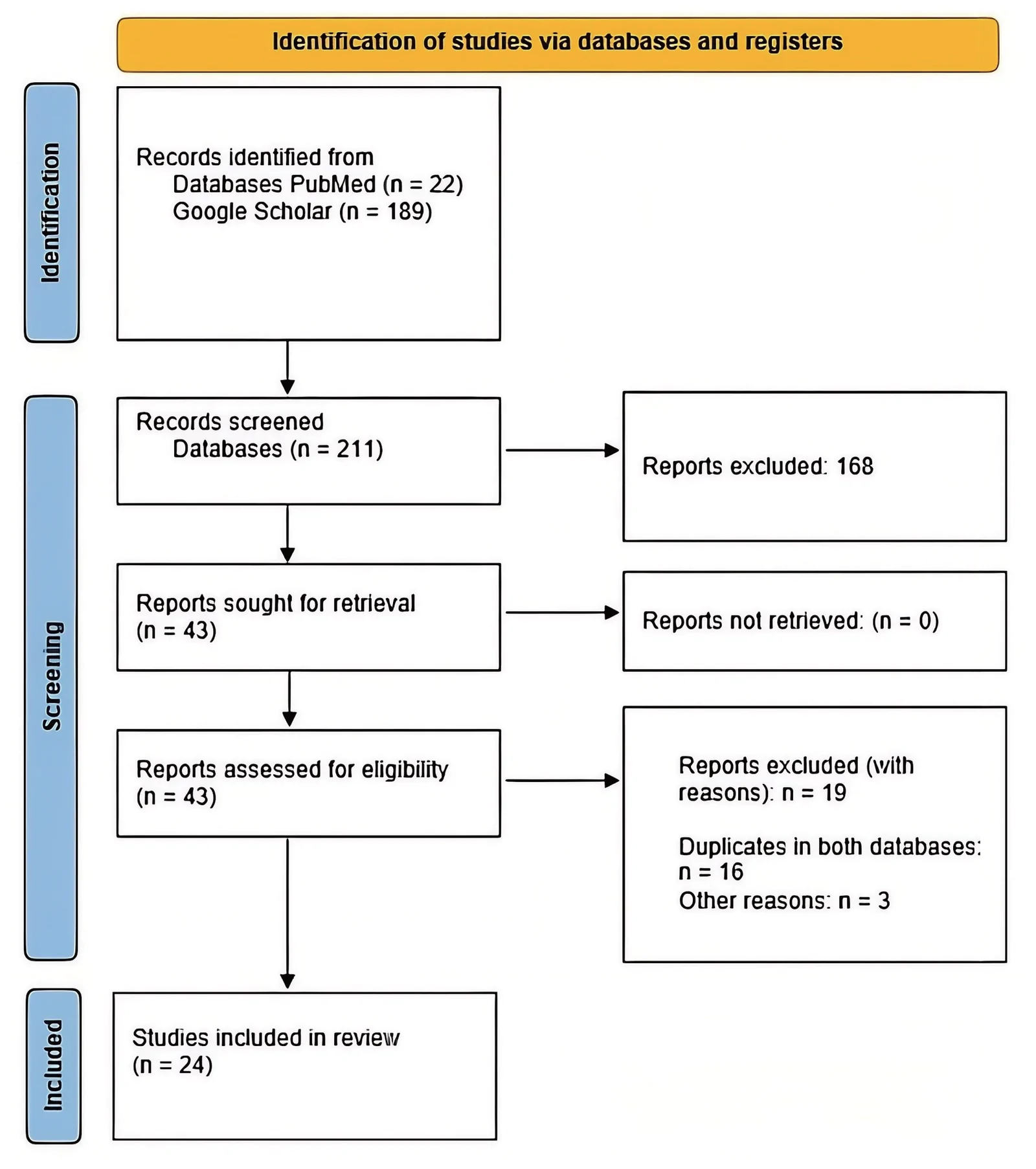
Flow diagram of study selection for enzyme-treated soybean meal (ESBM) in weanling pigs [21,46].

**Figure 2:**
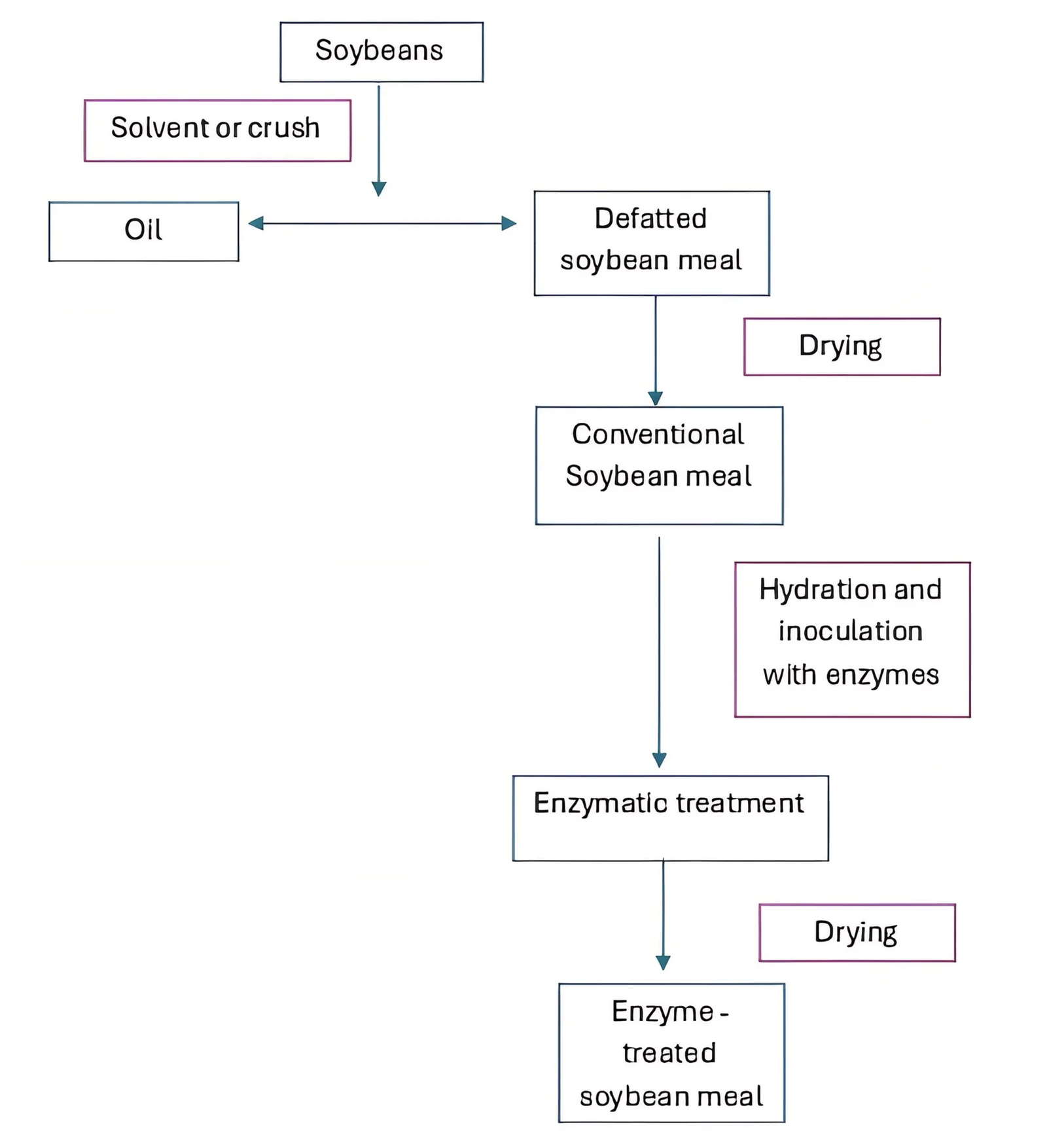
Processes involved in producing soybean meal and enzyme-treated soybean meal.

**Table 1:** Articles reporting results of experiments in which enzyme-treated soybean meal (ESBM) was included in diets for weanling pigs

Author	Publication	Exposure	Outcomes/conclusions	Search
Tan et al. [26]	Enzymolytic soybean meal-impact on growth performance, nutrient digestibility, antioxidative capacity, and intestinal health of weaned piglets	ESBM composition	Amino acids, small peptides, maltose, sucrose, raffinose, stachyose, starch, glycinin, and β-conglycinin. Inclusion levels of ESBM: 2%, 4%, and 6%	PubMed, Google Scholar
		Digestibility	Apparent ileal digestibility of dry matter, gross energy, crude protein, and ether extract in weaned piglets.	
		Growth performance/gut health	Substitution of soybean meal with ESBM resulted in growth improvement in weaned pig, attributed to enhanced nutrient digestibility, antioxidant capacity, and intestinal barrier function.	
Tang et al. [7]	Effect of heating, microbial fermentation, and enzymatic hydrolysis of soybean meal on growth performance, nutrient digestibility, and intestinal microbiota of weaned piglets	ESBM composition	Lectins, glycinin, and β-conglycinin. Inclusion levels of ESBM: 17%	PubMed, Google Scholar
		Digestibility	Apparent total tract digestibility of energy and dry matter.	
		Growth performance/gut health	Dietary supplementation with ESBM reduced the diarrhea index and improved nutrient digestibility.	
Mallea et al. [25]	Nutritional value of a new source of cheese coproduct fed to weanling pigs	ESBM composition	Dry matter, crude protein, acid-hydrolyzed ether extract, ash, amino acids, and total dietary fiber. Inclusion levels of ESBM: 36% and 21.15%	PubMed, Google Scholar
		Digestibility	Apparent ileal digestibility and standardized ileal digestibility of crude protein and amino acids. Apparent total tract digestibility of dry matter and energy.	
Garavito-Duarte et al. [24]	Nutritional value of high protein ingredients fed to growing pigs in comparison to commonly used protein sources in swine diets	ESBM composition	Dry matter, crude protein, crude fiber, ash, amino acids. Inclusion level of ESBM: 28%	PubMed, Google Scholar
		Digestibility	Apparent ileal digestibility of amino acids. Standardized ileal digestibility of amino acids.	
Deng et al. [40]	Comparative effects of soy protein concentrate, enzyme-treated soybean meal, and fermented soybean meal replacing animal protein supplements in feeds on growth performance and intestinal health of nursery pigs	ESBM composition	Glycinin and β-conglycinin. Inclusion levels of ESBM: 33%, 67%, and 100%.	PubMed
		Digestibility	Apparent ileal digestibility of dry matter, crude protein, and energy.	
		Growth performance/gut health	Soy protein concentrate, enzyme-treated soybean meal, and fermented soybean meal reduced the use of animal protein supplements up to 33% until 7 kg, up to 67% from 7 kg to 11 kg, and entirely after 11 kg without affecting intestinal health or growth performance.	
Yang et al. [27]	Comparative efficacy of fish meal replacement with enzymatically treated soybean meal on growth performance, immunity, oxidative capacity and fecal microbiota in weaned pigs	ESBM composition	Dry matter, crude protein, ash, amino acids, trypsin inhibitors, glycinin, and β-conglycinin. Inclusion levels of ESBM: 11.96% and 15.26%	PubMed, Google Scholar
		Growth performance/gut health	Replacing fish meal with enzymatically treated soybean protein shows equal or better growth performance, accompanied by a decreasing diarrhea rate. ESBM has a positive impact on serum concentrations of indicators for intestinal integrity, and antioxidant capacity.	
Engelsmann et al. [23]	Age-dependent development in protein digestibility and intestinal morphology in weaned pigs fed different protein sources	Digestibility	Standardized ileal digestibility of crude protein and amino acids	PubMed, Google Scholar
		Growth performance/gut health	No differences between wheat, soybean meal, ESBM, rapeseed meal, and casein were found in terms of morphological characteristics of the small intestine or incidence of diarrhea.	
Zhang and Piao [31]	Different dietary protein sources influence growth performance, antioxidant capacity, immunity, fecal microbiota, and metabolites in weaned piglets	ESBM composition	Dry matter, crude protein, crude fiber, lysine, methionine, threonine, tryptophan, calcium, phosphorus, starch. Inclusion levels of ESBM: 7.5% and 15.7%	PubMed, Google Scholar
		Digestibility	Apparent total tract digestibility of crude protein and gross energy	
		Growth performance/gut health	ESBM increased average daily gain and improved antioxidant status and immunity via increased serum levels and modulated fecal microbiota composition because of reduced protein fermentation.	
Li et al. [14]	Enzyme-treated soybean meal replacing extruded full-fat soybean affects nitrogen digestibility, cecal fermentation characteristics, and bacterial community of newly weaned piglets	ESBM composition	Dry matter, crude protein, ether extract, ash, gross energy, neutral detergent fiber, acid detergent fiber, trypsin inhibitors, glycinin and β-conglycinin Inclusion levels of ESBM: 7%	PubMed, Google Scholar
		Digestibility	Apparent ileal digestibility of amino acids	
		Growth performance/gut health	ESBM improved nitrogen utilization and inhibited protein fermentation in the hindgut of weaned piglets.	
Long et al. [29]	Enzyme-treated soybean meal enhanced performance via improving immune response, intestinal morphology and barrier function of nursery pigs in antibiotic free diets	Growth performance/gut health	ESBM enhances growth performance via improving antioxidant status, immune response, intestinal morphology, and barrier function of nursery pigs. Inclusion levels of ESBM: 14%	PubMed, Google Scholar
Acosta et al. [28]	Corn protein has greater concentrations of digestible amino acids and energy than low-oil corn distillers dried grains with soluble when fed to pigs but does not affect the growth performance of weanling pigs	ESBM composition	Dry matter, crude protein, acid-hydrolyzed ether extract, ash, and amino acids.	PubMed, Google Scholar
Nørgaard et al. [30]	Amino acid absorption profiles in growing pigs fed different protein sources.	ESBM composition	Dry matter, crude protein, amino acids, and total dietary fiber. Inclusion levels of ESBM: 34.8%	Google Scholar
Ruckman et al. [19]	The effects of enzymatically treated soybean meal on growth performance and intestinal structure, barrier integrity, inflammation, oxidative status, and volatile fatty acid production of nursery pigs	Growth performance/gut health	ESBM improved oxidative status and intestinal barrier integrity, but impact on intestinal inflammation and morphology was minimal. Inclusion levels of ESBM: 7%, 14%, and 21%	PubMed, Google Scholar
Ton Nu et al. [34]	Thermomechanical and enzyme-facilitated processing of soybean meal enhanced in vitro kinetics of protein digestion and protein and amino acid digestibility in weaned pigs	ESBM composition	Dry matter, crude protein, crude fiber, ash, amino acids, calcium, phosphorus, starch, neutral detergent fiber, acid detergent fiber, trypsin inhibitors, lectins, glycinin, and β-conglycinin	Google Scholar
		Digestibility	Apparent ileal digestibility of crude protein and amino acids	
Li et al. [37]	Effects of different protein sources completely replacing fish meal in a low-protein diet on growth performance, intestinal digestive physiology, and nitrogen digestion and metabolism in nursery pigs	ESBM composition	Dry matter, crude protein, crude fiber, ether extract, ash, amino acids, calcium, phosphorus, starch, trypsin inhibitors, glycinin, and β-conglycinin Inclusion levels of ESBM: 4%	PubMed, Google Scholar
		Digestibility	Apparent ileal digestibility of crude protein and amino acids.	
		Growth performance/gut health	ESBM replaced fish meal in low-protein, amino acids-supplemented diets without affecting growth performance and maintained intestinal digestive physiology, nitrogen digestion and metabolism in nursery pigs. Feeding pigs with HP300 decreased fecal nitrogen excretion.	
Ma et al. [18]	Comparative effects of enzymolytic soybean meal and antibiotics in diets on growth performance, antioxidant capacity, immunity, and intestinal barrier function in weaned pigs	ESBM composition	Dry matter, crude protein, ether extract, ash, amino acids, gross energy, sucrose, raffinose, verbascose, neutral detergent fiber, acid detergent fiber, trypsin inhibitors, glycinin, and β-conglycinin Inclusion levels of ESBM: 9% and 7.5%	Google Scholar
		Digestibility	Apparent total tract digestibility of dry matter, crude protein, and gross energy.	
		Growth performance/gut health	ESBM reduced diarrhea incidence and improved growth performance by improving antioxidant capacity, immunity, and intestinal barrier function.	
Ma et al. [16]	Effects of replacing soybean meal, soy protein concentrate, fermented soybean meal or fish meal with enzyme-treated soybean meal on growth performance, nutrient digestibility, antioxidant capacity, immunity, and intestinal morphology in weaned pigs.	ESBM composition	Dry matter, crude protein, ether extract, ash, amino acids, gross energy, sucrose, raffinose, verbascose, neutral detergent fiber, acid detergent fiber, trypsin inhibitors, glycinin, and β-conglycinin. Concentration of small peptides.	Google Scholar
		Growth performance/gut health	ESBM improved growth performance, immune function, and antioxidant capacity as effectively as fish meal. ESBM could replace soybean meal, soy protein concentrate, fermented soybean meal or fish meal in weaned pig diets. ESBM could be used in weaned pig diets for at least 28 days.	
Jones et al. [36]	Evaluating the effects of fish meal source and level on growth performance of nursery pigs	ESBM composition	Dry matter, crude protein, ether extract, ash, amino acids, calcium, and phosphorus	PubMed, Google Scholar
Navarro et al. [11]	Amino acid digestibility by weanling pigs of processed ingredients originating from soybeans, 00-rapeseeds, or a fermented mixture of plant ingredients	ESBM composition	Dry matter, crude protein, acid-hydrolyzed ether extract, ash, amino acids, calcium, phosphorus, energy, sucrose, raffinose, stachyose, neutral detergent fiber, acid detergent fiber, and trypsin inhibitors.	PubMed, Google Scholar
		Digestibility	Apparent ileal digestibility of crude protein, ash, and amino acids. Standardized ileal digestibility of crude protein and amino acids.	
Hossain et al. [35]	Apparent total tract digestibility and ileal digestibility of dry matter, nitrogen, energy, and amino acids in conventional, Bacillus subtilis-fermented, and enzyme-treated soybean meal fed to weanling pigs.	ESBM composition	Dry matter, crude protein, crude fiber, lysine, methionine, threonine, Tryptophan, calcium, phosphorus, starch, ether extract, ash, amino acids, calcium, phosphorus,	Google Scholar
		Digestibility	Apparent ileal digestibility of dry matter, crude protein, energy, and amino acids. Standardized ileal digestibility of crude protein and amino acids.	
Haghbayan and Shamsaie Mehrgan [45]	The effect of replacing fish meal in the diet with enzyme-treated soybean meal (HP310) on growth and body composition of rainbow trout fry	ESBM composition	Crude protein, crude fiber, ether extract, ash, amino acids, trypsin inhibitors, and phytic acid.	Google Scholar
Goebel and Stein [12]	Phosphorus digestibility and energy concentration of enzyme-treated and conventional soybean meal fed to weanling pigs	ESBM composition	Dry matter, crude protein, ether extract, ash, amino acids, calcium, phosphorus, sucrose, raffinose, stachyose, neutral detergent fiber, acid detergent fiber, trypsin inhibitors, glycinin, and β-conglycinin	PubMed, Google Scholar
		Digestibility	Apparent ileal digestibility of dry matter, ash, gross energy, crude protein, and amino acids. Standardized ileal digestibility of crude protein and amino acids.	
Zhou et al. [8]	Effect of feeding enzymolytic soybean meal on performance, digestion, and immunity of weaned pigs	ESBM composition	Dry matter, crude protein, ash, small peptide concentration, and trypsin inhibitors. Inclusion levels of ESBM: 5%, 10%, and 15%	Google Scholar
		Growth performance/gut health	Enzymolytic soybean meal partially replacing common soybean meal and soy protein isolate improved feed intake, weight gain, and immune function of weaned piglets.	
Cervantes-Pahm and Stein [6]	Ileal digestibility of amino acids in conventional, fermented, and enzyme-treated soybean meal and in soy protein isolate, fish meal, and casein fed to weanling pigs	ESBM composition	Dry matter, crude protein, crude fiber, ether extract, amino acids, calcium, phosphorus, glucose, fructose, raffinose stachyose, trypsin inhibitors, glycinin, and β-conglycinin	PubMed, Google Scholar
		Digestibility	Apparent ileal digestibility of dry matter, crude protein, and amino acids. Standardized ileal digestibility of crude protein and amino acids.	

**Table 2:** Concentrations of dry matter, fat, crude protein, amino acids (AA), ash, calcium, and phosphorus in soybean meal (SBM) and enzyme-treated soybean meal (ESBM), as-is basis

Item	n	SBM	SD	CV	n	ESBM	SD	CV
Dry matter (%)	9	89.00	1.32	1.49	22	92.11	1.84	2.00
Ether extract (%)	4	1.31	0.55	42.18	11	1.68	0.59	35.34
Acid-hydrolyzed ether extract (%)	2	2.40	1.65	68.79	4	1.70	0.64	37.70
Crude protein (%)	11	46.50	1.72	3.69	23	54.25	2.27	4.18
Indispensable AA (%)								
Arginine	11	3.36	0.30	8.91	20	3.93	0.81	20.48
Histidine	11	1.19	0.10	8.67	18	1.59	0.44	27.43
Isoleucine	11	2.15	0.30	13.72	20	2.57	0.52	20.23
Leucine	11	3.61	0.30	8.24	20	4.25	0.82	19.22
Lysine	11	2.94	0.20	6.80	22	3.45	0.67	19.30
Methionine	11	0.63	0.07	11.74	21	0.69	0.22	31.80
Phenylalanine	11	2.53	0.66	26.15	20	3.04	0.89	29.41
Threonine	11	1.81	0.13	7.23	21	2.19	0.42	19.11
Tryptophan	8	1.35	1.92	141.58	17	0.70	0.17	23.54
Valine	11	2.23	0.30	13.27	20	2.68	0.55	20.69
Dispensable AA (%)								
Alanine	9	1.96	0.22	11.27	18	2.31	0.23	9.79
Aspartic acid	10	5.34	0.43	8.02	18	5.91	0.48	8.08
Cysteine	11	0.76	0.23	30.04	18	0.79	0.21	26.52
Glutamic acid	10	8.54	0.64	8.62	18	9.36	0.58	6.22
Glycine	10	1.96	0.16	8.30	18	2.35	0.38	16.12
Proline	10	2.33	0.22	9.54	18	2.69	0.23	8.49
Serine	10	2.22	0.32	14.63	18	2.46	0.28	11.54
Tyrosine	9	1.59	0.23	14.18	18	1.87	0.25	13.49
Ash (%)	8	6.33	0.47	7.47	19	6.79	0.54	7.90
Calcium (%)	5	0.28	0.05	17.00	13	0.32	0.04	13.79
Phosphorus (%)	5	0.64	0.03	4.89	13	0.72	0.03	3.92
Phytate phosphorus (%)	1	0.48	-	-	1	0.11	-	-

Values obtained from references included in this review.

SD, standard deviation; CV, coefficient of variation.

**Table 3:** Concentrations of energy, carbohydrates, and anti-nutritional factors in conventional soybean meal (SBM) and enzyme-treated soybean meal (ESBM), as-is basis

Item	n	SBM	SD	CV	n	ESBM	SD	CV
Energy (kcal/kg)								
Gross energy	4	4,203	72.58	1.73	6	4,493	54.15	1.21
Digestible energy	-	-	-	-	1	3,887	-	-
Metabolizable energy	-	-	-	-	1	3,634	-	-
Glucose (%)	-	-	-	-	1	0.49	-	-
Maltose (%)	1	0.45	-	-	1	0.01	-	-
Fructose (%)	1	0.63	-	-	1	1.11	-	-
Sucrose (%)	6	5.63	1.53	27.27	8	1.15	1.68	146.63
Starch (%)	2	1.83	0.09	4.72	4	1.02	0.41	40.56
Crude fiber (%)	5	3.93	1.79	45.48	10	3.84	0.97	25.19
Neutral detergent fiber (%)	5	9.97	3.83	38.43	9	11.92	2.38	19.96
Acid detergent fiber (%)	5	6.17	1.93	31.33	9	5.74	1.01	17.62
Total dietary fiber (%)	1	20.30	-	-	2	21.02	3.82	18.17
Insoluble dietary fiber (%)	1	17.50	-	-	2	18.97	2.37	12.47
Soluble dietary fiber (%)	1	2.80	-	-	2	2.06	1.46	70.80
Raffinose (%)	5	1.08	0.10	9.42	9	0.49	0.60	122.28
Stachyose (%)	5	4.29	1.31	30.51	9	0.81	0.80	99.54
Anti-Nutritional factors								
Trypsin inhibitors (TIU/mg)	6	5.58	2.62	46.86	15	1.71	0.86	50.12
Lectins (mg/kg)	1	300.00	-	-	1	29.00	-	-
Glycinin (mg/g)	6	104.86	161.75	154.25	13	6.96	15.38	221.04
β-Conglycinin (mg/g)	6	40.04	57.54	143.74	13	1.22	2.11	172.64

Values obtained from references included in this review.

SD, standard deviation; CV, coefficient of variation.

**Table 4:** Peptide size of protein in soybean meal (SBM) and enzyme-treated soybean meal (ESBM), as-feed basis

Peptide size concentration (g/100 g of crude protein)	SBM	ESBM
>60 kDa	27.00	28.87
20–60 kDa	43.16	37.51
<20 kDa	29.84	33.62
Small peptide<500 Da	3.66	18.30

Values obtained from references included in this review [6,16].

**Table 5:** Apparent ileal digestibility of dry matter, ash, gross energy, crude protein, and amino acids (AA) in enzyme-treated soybean meal

Item	NRC [10]	Tan et al. [26]	Garavito-Duarte et al. [24]	Mallea et al. [25]	Li et al. [14]	Ton Nu et al. [34]	Li et al. [15]	Goebel and Stein [12]	Goebel and Stein [12]	Hossain et al. [35]	Cervantes-Pahm and Stein [6]	Average	SD	CV
Dry matter (%)	-	-	54.5	-	-	-	-	-	-	82.6	61.3	66.1	10.4	15.7
Crude protein (%)	82.0	72.8	61.7	-	-	84.2	83.2	81.9	75.1	83.0	76.8	77.3	6.7	8.7
Ash (%)	-	-	-	-	-	-	-	37.9	37.2	-	-	37.5	0.3	0.8
Gross energy (%)	-	-	55.6	-	-	-	-	-	-	81.7	-	68.6	10.6	15.5
Indispensable AA (%)														
Arginine	92.0	88.9	-	92.5	75.1	93.1	89.0	91.6	87.4	81.0	90.4	87.7	5.3	6.1
Histidine	87.0	81.6	-	91.9	88.3	89.2	83.9	89.4	86.1	80.7	82.7	86.0	3.5	4.1
Isoleucine	86.0	81.4	-	92.0	70.7	88.8	85.1	88.7	85.3	83.3	84.4	84.4	5.4	6.4
Leucine	86.0	83	-	91.7	69.8	88.4	82.0	88.5	85.7	80.5	83.7	83.7	5.6	6.7
Lysine	83.0	76.6	-	87.3	85.0	89.3	85.8	84.5	79.1	77.9	82.2	83.1	3.9	4.7
Methionine	88.0	83.4	-	93.0	86.5	91.0	89.1	90.3	87.6	84.6	86.2	88.0	2.8	3.2
Phenylalanine	83.0	83.5	-	92.3	92.1	89.2	85.2	89.6	86.6	83.9	85.5	87.5	3.0	3.4
Threonine	78.0	69	-	85.6	74.9	81.7	79.1	80.7	76.5	77.2	72.5	77.5	4.5	5.8
Tryptophan	80.0	79.4	-	90.7	77.6	90.3	83.1	89.5	85.8	-	82.1	84.8	4.5	5.3
Valine	84.0	76.9	-	88.8	73.8	85.5	83.2	85.3	80.7	84.6	78.9	82.0	4.3	5.2
Dispensable AA (%)														
Alanine	82.0	72.6	-	85.9	66.0	85.1	79.1	81.1	75.8	76.7	77.3	77.7	5.5	7.1
Aspartic acid	83.0	76.7	-	88.6	80.9	86.2	82.0	85.3	82.6	79.7	82.4	82.7	3.2	3.9
Cysteine	68.0	52.2	-	79.6	75.6	76.5	74.0	74.8	69.2	78.2	73.7	72.6	7.3	10.1
Glutamic acid	86.0	80.5	-	91.4	81.9	89.4	83.0	87.6	84.3	81.7	89.3	85.5	3.6	4.2
Glycine	76.0	56	-	71.5	73.4	78.4	63.8	64.6	52.5	73.6	59.3	65.9	7.9	12.0
Proline	73.0	74.8	-	39.5	78.8	75.8	79.8	31.0	−0.2	80.4	43.9	63.0	18.5	29.4
Serine	83.0	76.7	-	89.6	77.3	86.4	84.3	86.7	83.6	82.0	78.9	82.8	4.0	4.8
Tyrosine	86.0	81.1	-	89.9	71.9	89.1	85.2	89.2	85.7	88.0	86.0	85.1	5.1	5.9

SD, standard deviation; CV, coefficient of variation.

**Table 6:** Standardized ileal digestibility of crude protein and amino acids (AA) in enzyme-treated soybean meal

Item	Tan et al. [26]	Mallea et al. [25]	Ton Nu et al. [34]	Goebel and Stein [12]	Goebel and Stein [12]	Hossain et al. [35]	Cervantes-Pahm and Stein [6]	NRC [10]	Average	SD	CV
Crude protein (%)	82.0	-	-	89.9	85.2	88.9	91.9	88.0	87.6	3.5	4.0
Indispensable AA (%)											
Arginine	92.8	99.6	96.0	96.9	93.5	84.1	98.1	96.0	94.4	4.8	5.1
Histidine	86.5	95.5	92.6	93.4	90.6	84.5	88.8	90.0	90.3	3.6	4.0
Isoleucine	86.3	95.1	91.9	91.7	88.9	86.3	89.7	89.0	90.0	3.0	3.3
Leucine	86.6	94.9	91.6	91.7	89.2	83.9	89.2	89.0	89.6	3.4	3.7
Lysine	81.1	89.4	92.3	87.3	82.5	82.0	88.1	86.0	86.1	3.9	4.6
Methionine	87.7	95.9	93.6	92.9	90.5	88.8	92.1	91.0	91.6	2.6	2.9
Phenylalanine	87.9	95.3	91.9	92.6	90.1	86.6	91.8	86.0	90.9	2.7	3.0
Threonine	79.5	89.5	88.4	87.5	83.9	83.1	85.7	83.0	85.4	3.2	3.8
Tryptophan	85.4	93.8	94.0	93.3	90.0	-	87.5	83.0	90.7	3.3	3.7
Valine	83.4	92.6	90.3	90.0	86.2	89.3	89.4	89.0	88.7	2.8	3.2
Dispensable AA (%)											
Alanine	80.1	90.6	90.1	88.3	83.9	85.7	88.5	86.0	86.7	3.5	4.0
Aspartic acid	80.8	91.1	89.2	88.5	86.3	83.5	88.1	86.0	86.8	3.3	3.8
Cysteine	63.6	82.1	84.5	82.3	77.3	85.1	85.1	73.0	80.0	7.2	8.9
Glutamic acid	83.8	93.7	91.6	90.1	87.1	85.9	93.5	88.0	89.4	3.6	4.0
Glycine	71.9	78.0	88.4	86.6	77.7	87.4	94.4	89.0	83.5	7.2	8.7
Proline	96.1	53.1	97.8	101.2	80.3	87.3	148.2	112.0	94.8	26.5	27.9
Serine	84.3	94.1	91.4	92.0	89.2	87.9	89.2	87.0	89.7	2.9	3.3
Tyrosine	86.7	92.8	92.5	92.4	89.5	92.3	92.0	92.0	91.2	2.1	2.3

SD, standard deviation; CV, coefficient of variation.

**Table 7:** Apparent total tract digestibility of dry matter, crude protein, ether extract, gross energy, calcium, and phosphorus in enzyme-treated soybean meal

Item	Tan et al. [26]	Tan et al. [26]	Tan et al. [26]	Tang et al. [7]	Tan et al. [26]	Ma et al. [18]	Hossain et al. [35]	Goebel and Stein [12]	Goebel and Stein [12]	Goebel and Stein [12]	Average	SD	CV
Dry matter (%)	82.7	85.7	82.0	86.8	86.4	81.5	88.9	-	96.4	96.3	87.3	5.3	6.1
Crude protein (%)	78.9	79.6	81.8	72.5	-	76.0	88.8	-	-	-	79.6	5.1	6.4
Ether extract (%)	64.1	71.0	61.2	-	-	-	-	-	-	-	65.4	4.1	6.3
Gross energy (%)	82.2	84.9	80.9	82.3	88.3	81.9	87.7	87.6	88.0	-	84.6	2.7	3.2
Calcium (%)	-	-	-	-	-	-	-	-	53.6	63.8	58.7	5.1	8.7
Phosphorus (%)	-	-	-	-	-	-	-	-	59.8	63.8	61.8	2.0	3.2

SD, standard deviation; CV, coefficient of variation.

## Data Availability

Upon reasonable request, the datasets of this study can be available from the corresponding author.
